# Learning to play to learn in pediatric physical therapy

**DOI:** 10.3389/fpsyg.2024.1467323

**Published:** 2025-01-06

**Authors:** Ragnhild B. Håkstad, Stacey C. Dusing, Gay L. Girolami, Gunn Kristin Øberg, Hanne De Jaegher

**Affiliations:** ^1^Department of Health and Care Sciences, Faculty of Health, UiT The Arctic University of Norway, Tromsø, Norway; ^2^Motor Development Lab, Department of Biokinesiology and Physical Therapy, University of Southern California, Los Angeles, CA, United States; ^3^Department of Physical Therapy, University of Illinois at Chicago, Chicago, IL, United States; ^4^Chat Lab, Department of Psychology, University of Sussex, Brighton, United Kingdom

**Keywords:** pediatric physical therapy, play, enactive theory, qualitative, interview, motor learning

## Abstract

**Introduction:**

Play is a way for children to develop and learn about themselves in conjunction with the world. Using play as part of pediatric physical therapy is broadly recommended. This study investigates this integration of play and seeks to answer the research question: How do pediatric physical therapists (PPT) understand and manage embedding play in pediatric physical therapy with children aged 0–3?

**Methods:**

This is a qualitative study in which we connect to an enactive theoretical framework. We interviewed 14 PPTs about their use of play, including video-elicited questions while viewing recordings of their therapy sessions. Our results were developed through an abductive thematic analysis.

**Results:**

The PPTs acknowledge play as a foundation of children’s learning and a vital component of physical therapy. They explain that play and therapy often co-exist and intertwine, but they also experience tensions when they strive to make play therapeutic. The PPTs find it taxing to engage in play with children who present with limited interaction and play skills, and voice concern for children who struggle to engage in interactional play.

**Discussion:**

Trusting play and letting play emerge through shared sense-making can resolve challenges and enable PPTs to discover new therapeutic opportunities. A child’s striving and overcoming of resistance can be infused with playfulness and make play thrive. We invite PPTs to experiment with the emerging opportunities and boundaries between therapy and play during treatment sessions. Respect for the child’s autonomy, attention to the child’s play experience, and repairs of interactional mismatches are crucial in this process. Therapeutic guidance and mutuality in interactions can empower children to learn to play to learn new skills and experience mastery as they explore and venture beyond what they already know.

## Introduction

Play is a multi-faceted phenomenon that has been defined and researched from different perspectives over time. In this paper, we will focus on the dimensions of play that are most relevant for the play of young children aged 0–3, namely play as a way to develop and learn about oneself in conjunction with the world. Our play perspective aligns with the consensus definition developed by Fiss et al. (2023): “We define play as an active process by which an individual is intrinsically motivated to explore the self, the environment, and/or interactions with another person. It is enjoyable with a natural flow individually or between participants. Play is valued for its own sake; the means are more valuable than the ends.”

An important and debated question is how play relates to learning and development. Our position is that play is an activity that should be valued for its own sake, but at the same time an important driver for learning and not just a behavior of abundance and recreation. Play inhabits crucial learning elements such as motivation, engagement, exploration, repetition, and variation (Fiss et al., 2023; Herzberg et al., 2022; Lobo et al., 2014). Accordingly, we consider play to be essential for development across motor, cognitive, social–emotional, and adaptive domains (Ginsburg et al., 2007; Lifter et al., 2011; Yogman et al., 2018). In play, children learn to move their bodies and expand their minds (Henricks, 2015a). They learn to interact with and adapt their behavior to the physical and social world in which they are playing, and they learn to deal with emotions, both their own and those of their interaction partners (Ginsburg et al., 2007; Lifter et al., 2011; Yogman et al., 2018).

Using play as part of pediatric physical therapy is broadly recommended (Fiss et al., 2023; Lifter et al., 2011; Lifter et al., 2011). However, pediatric physical therapy is more than just play and play is generally not taught in physical therapy curricula. Children with motor disabilities receive physical therapy to improve their motor abilities (Inamdar et al., 2021). Setting goals and structuring activities to achieve these goals is at the core of pediatric physical therapy practice (Pritchard-Wiart et al., 2019; Bexelius et al., 2018). This challenges the idea of embedding and applying play during therapy as an activity that is valued for its own sake. Moreover, it questions the perspective that pediatric physical therapists (PPT) can have an impact on play which could be a goal itself or that play could be a tool by which therapists can address other goals. Research on embedding play in pediatric physical therapy was recently summarized by Fiss et al. (2023). They highlight the importance of engaging the child in play and adapting therapeutic play to the individual child’s needs and abilities. Play activities that facilitate development across domains are recommended, and both novel experiences and managing the “just right” level of challenges are essential for the upholding of a child’s engagement. In the work to achieve therapeutic goals, an important therapeutic task is to simultaneously create an environment that provides ample opportunities for exploration and interaction and allows the child to be an initiator of play.

In previous research, Håkstad et al. (2017) investigated the benefits of integrating play in physical therapy sessions with preterm infants 3–14 months old. They established the concept “enactive therapeutic sensory-motor play” and highlighted that “therapeutic actions and handling, choices of toys and changes to the task or environment all need to be part of the game, not a disturbance to it” (Håkstad et al., 2017). For this play to be therapeutic, PPTs need to “continuously address the infant’s specific motor impairments and facilitate improvements to the infant’s motor performance” (Håkstad et al., 2017). The more recent study by Håkstad et al. (2022) explored how bodily interactions and subtle, responsive therapeutic handling supported young children’s playing-to-learn-to-move processes; i.e., enabled them to explore and discover new movement solutions during play. To date, research on PPTs’ own perceptions on embedding play as part of physical therapy is lacking. In this current study we explore PPTs’ explanations and experiences from clinical practice and seek to develop new knowledge about their reasoning and interaction processes by answering the research question: How do PPTs understand and manage embedding play in pediatric physical therapy with children aged 0–3?

### An enactive approach

The enactive approach (Varela et al., 2016) is the theoretical framework for the study. This approach integrates knowledge from neuroscience, dynamic systems theory, and phenomenology to understand human embodied, social cognition and how we enact our worlds. Enactive theory builds on five core terms: autonomy, embodiment, experience, emergence, and sense-making (Varela et al., 2016). Autonomy refers to individuals’ active generation and maintenance of their own identity in interaction with their world (Di Paolo, 2005; Thompson, 2007). The term applies from “cell to society” (Froese and Di Paolo, 2011), i.e., from the basic biological upholding of life to complex behaviors in social contexts. Embodiment refers to the way individuals, as particular, material living bodies, care for, act in, and interact with the world out of their specific, concrete bodily and situated needs and constraints in continuous brain–body–environment interactions (Froese and Di Paolo, 2011). Experience is what molds and transforms us as individuals (Froese and Di Paolo, 2011). It is through our experiences in the world that we learn to cognize about it and ourselves in it. In this paper, we focus on the terms sense-making and emergence because we found them particularly helpful in our interpretations of the PPTs’ perspectives on play and clinical reasoning in pediatric physical therapy.

Sense-making is our always ongoing activity of experiencing and making meaning in and of our interactions with the world. It is participatory and intersubjective; we understand our world through our social interactions in it (De Jaegher and Di Paolo, 2007). As sense-makers, each person has their intentions that they bring into their social interactions with others. These intentions are based on the cares, needs, and constraints of each particular, bodily and situated person, and shape their priorities and perception of significant events during interaction (De Jaegher and Di Paolo, 2007). For example, an important intention of the PPTs is to provide therapy that effectively supports children’s development and learning. It is an articulate intention that is driven by the professional role they want to fulfill. Young children’s intentions will typically be less articulate but are generally expressed through their behavior, play, and interactional engagements with people and surroundings.

During interactions, shared intentions provide common ground for action, while individual intentions may produce counteractive behaviors or may develop into shared intentions (De Jaegher and Di Paolo, 2007; Fantasia et al., 2014). All this happens through participatory sense-making processes in which the interactors move between their own behaviors and intentions, those of their interactional partner, and those emerging between them (De Jaegher and Di Paolo, 2007). Making sense together is an ongoing, flexible, and sometimes unpredictable process of moving from tensions emerging between individual intentions, to resolutions of these tensions, and on to new tensions (De Jaegher and Di Paolo, 2007).

Clinical reasoning is a specific form of sense-making, informed by professional background and knowledge (Øberg et al., 2015; Kirk-Sanchez et al., 2022; Higgs et al., 2019). However, this sense-making is also influenced by the ongoing interactions and cooperation between the PPT and child (Håkstad et al., 2018). During play and therapy, the PPT and child cooperate on a shared activity in which they both contribute with and move between intentions. It is a cooperative setting that shapes the clinical reasoning and choices of actions of the PPT, and at the same time shapes the child’s sense-making and learning of both motor and cooperation skills (Fantasia et al., 2014).

Emergence seeks to explain how novel behaviors form and develop through our interactions with our physical and social environments (Froese and Di Paolo, 2011). In interactions in which the participants are mutually engaged and influencing the ongoing sense-making, what emerges between the participants is mutually shaped and developed by the balanced interaction between the interactors (Fuchs and De Jaegher, 2009). Whereas in less mutual interactions, one participant will to a larger extent seek to influence what emerges in the interactions between the two (Fuchs and De Jaegher, 2009). The agency of each participant thus relies on regulation between the interactors, in which they may complement, compete, enter into unavoidable tensions between their intentions, resolve these tensions and move into new ones. What emerges depends on what we encounter as we interact with others and our worlds, and the dynamics of the interaction process itself co-determine how the participants engage and regulate themselves to each other (De Jaegher et al., 2010).

In PPTs’ practice, it is the ongoing play, the PPT’s therapeutic agenda, and the child’s intentions, wishes, cares and concerns, and the interaction that emerges between them, that together influence how actions, interactions, and sense-making emerge between the child and PPT. One pertinent question in this regard is to what extent the PPTs allow play to influence emerging actions and interactions with the child, and their flexibility with interactively emerging play. Similarly, what emerges during therapy will also depend on the ways and extent to which the child is able or willing to negotiate the premises of play and allow for the PPT’s integration of therapeutic elements into their play.

Connecting the enactive theoretical framework to play, children are autonomous beings who make sense of their embodied experiences and social encounters with others through play, and it is through these experiences that new skills emerge and develop. In brief, children learn to enact their world through play, and learning-to-move processes are typically also playing-to-learn-to-move processes in children’s everyday lives. This perspective underscores why play is an important piece of physical therapy as a means of facilitating motor learning and development.

## Materials and methods

### Study design

This is an explorative, interpretive qualitative study based on interviews with PPTs, including video-elicited questions related to selected clips from observations of their own treatment sessions with two children aged 0–3. We had an abductive approach in which the theoretical framework was planned at onset, but with an inductive first phase of analysis and more detailed decisions about which theoretical aspects to attend to as the analysis proceeded.

### Recruitment and participants

We recruited 14 PPTs via professional collaborators in the local communities in Norway and in the USA. In Norway, we also recruited via social media and newsletter through the Physical Therapy Union’s pediatric group. We searched for PPTs who had a particular interest in the use of play as part of their therapeutic approach. The PPTs gave their written informed consent to participate. They also shared information about the study with families on their caseload and informed the researcher about families who were willing to participate. The researcher provided families with additional information about the study and collected their informed consent. Information about participants were collected during interviews and in short questionnaires filled out by the PPTs and caregivers. All PPTs have post-educational training in pediatrics and clinical experience ranging from three to 30 years. The children in treatment were aged 0–3 and with a variety of diagnoses and functional levels ranging from mild motor delays to more severe developmental delays and medical co-conditions. An overview of participant demographics is provided in Tables 1, 2.

**Table 1:** Information about participating PPTs.

PPT	Years of experience	Years in pediatrics	Post-graduate pediatric education and courses
1	13	7.5y 100%	Various courses
2	26	23y 100%, 3y <50%	1-year post-education and various courses
3	23	21y 100%	Vojta training and various courses
4	–	20y <50%	Clinical specialist, psychodrama education
5	26	26y 100%	Various courses and conferences
6	3	3y 100%	Various courses
7	6.5	3y 100%, 2y >50%	Various courses
8	20	17y 100%	Various courses
9	26	20y 100%, 3y 50%	Various courses
10	8	1y 100%, 6y 50%	1-year post-education
11	7	2 y 100%, 5y 50%	Various courses & workshops
12	30	10100%, 20y 50%	NDT training
13	20	20 y 100%	Various courses and conferences
14	25	14y 100%, 9 y 50%	Clinical specialist
15	12	8 y 100%, 4 y 505	1-year post-education and various courses

**Table 2:** Information about participating children.

Child	Diagnosis^1^	Age at session	Motor function^2^	Developmental delay^3^
1	Unknown	1 year 7 months	Cruising, walking with support, handles toys.	Motor delay
2	Downs syndrome	4 months	No floor mobility, initial grasping skills.	Motor delay
3	Syndrome disorder	2 years 1 month	Cruising, walking with support, handles toys.	Motor and language delay
4	Sæthre-Chotzen syndrome	11 months	Cruising, walking with support, handles toys.	None of significance
5	Cerebral Palsy	1 year 6 months	Creeping/crawling, independent sitting, standing with support, handles toys.	Motor delay
6	Chromosome lesion disorder	8 months	No floor mobility, handles toys.	Motor delay
7	Unknown	3 years	Walking alone, handles toys.	Mild motor delay
8	Kidney disease	2 years	Walking alone, handles toys.	Mild motor delay
9	Cell migration disorder	1 year 6 months	Cruising, walking with support, handles toys.	Motor and language delay
10	Down syndrome	2 years	Walking alone, handles toys.	Motor, cognitive and language delay
11	Preterm	5 months (3,5 months CA^4^)	No floor mobility, initial grasping skills.	None of significance
12	Prader-Willi Syndrome, preterm	10 months	Crawling, sitting with support, handles toys.	Motor delay
13	Torticollis, moderate hearing loss	10 months	Creeping, cruising, handles toys.	None of significance
14	Unknown	2 years	Walking alone, handles toys.	Mild motor and language delay
15	Chromosome mutation disorder	2 years	No floor mobility, handles toys.	Severe motor and cognitive delay
16	Cerebral Palsy, hydrocephalus	11 months	Crawling, sitting and standing with support, handles toys.	Motor delay
17	Preterm	7 months (5 months corrected age)	No floor mobility, handles toys.	Motor delay
18	Cerebral Palsy	2 years	Crawling, sitting and standing with support, walking with assistive device, handles toys.	Motor delay
19	Cerebral Palsy	2 years	Crawling, sitting and standing with support, walking with assistive device, handles toys.	Motor delay
20	Lysosomal storage disease	2 years	Crawling, standing with support, handles toys.	Motor and language delay
21	Cerebral palsy	2 years	Walking alone, handles toys.	None of significance
22	Preterm	15 months (12 months corrected age)	Crawling, unsustained sitting, cruising, handles toys.	Motor and language delay
23	Unknown	1 year 18 months	Walking alone, handles toys.	Motor and language delay
24	Down syndrome	1 year 2 months	Sitting, cruising, handles toys.	Motor and language delay
25	Cerebral Palsy	2 years	Walking alone, handles toys.	None of significance
26	Spina bifida	14 months	Crawling, sitting, crouched standing with support, handles toys.	Motor delay
27	Unknown	2 years 6 months	Walking alone, handles toys.	Motor delay
28	Preterm	8 months (5 months CA)	Rolling prone to supine, unsustained sitting, handles toys.	Motor delay
29	Torticollis	5 months	Pivots in prone, handles toys.	None of significance
30	Preterm, dysmature, orthopedic condition	11 months (9 months CA)	Creeping, sitting, cruising, handles toys.	None of significance

1 For anonymity, rare conditions are not specified.

2 Descriptions based on motor performance during observation.

3 Description based on information from PPT/caregiver.

4 CA, Corrected Age.

### Data collection

The interviews were conducted 1 or 2 days after the observations of therapy sessions. We used a semi-structured interview guide beginning each interview with general questions about play in physical therapy. This was followed by questions about the treatment of the children in the observed sessions and the PPT’s description and intention of role of play during therapy with these children. Next, video-elicited questions (Henry and Fetters, 2012) gave the opportunity to discuss play events and interactional tensions that arise during play in more detail and allowed the PPTs to explain their perceptions, interpretations, and clinical reasoning in specific situations. The first author conducted the data collection.

### Data analysis

All interviews were transcribed in a close to verbatim manner, i.e., we preserved the meaning of statements but made the text reader-friendly by excluding unnecessary repetitions, stuttering, and half-word utterances. We did a thematic analysis similar to the descriptions of Braun and Clarke (Braun and Clarke, 2006), using NVivo software (RRID: SCR_014802) to code and sort data. With our research question in mind, we inductively approached the data and coded all relevant meaning units with a broad range of codes that preserved the meaning content of each unit. Next, we sorted the codes into a range of sub-themes, onwards into fewer and broader themes, and arrived at the two main themes as presented in our results (for an overview of meaning units, codes and themes view Supplementary material). The first author was responsible for coding and sorting in NVivo, and for drafting the presentations of results. All findings were discussed in the research group to apply theoretical perspectives and develop interpretations as a team.

### Methodological considerations

Selection bias is a pertinent concern in this study (Wu et al., 2016; Stige et al., 2009). Not all PPTs would be willing to let an unfamiliar researcher observe your skills and behaviors during therapy sessions. This is probably reflected in the study sample; all participants are experienced PPTs with post-educational training. The selection of children might also be biased due to the PPTs’ choice of the best-fitting candidates for playful therapy from their caseload. There are few children in our sample with severe motor, cognitive, communicative, or social impairments. We suspect that the PPTs find it more difficult to engage in play with these children and thus did not select them as potential participants. While playing with children with more severe impairments was a topic during the general conversations with the PPTs, this selection bias limited the opportunity to discuss such play interactions as part of the video-elicited questions.

In our dialogue with the participants, the PPTs confirmed that sessions for the most part proceeded as they normally would. However, involvement of parents and caregivers were most likely reduced due to the researcher’s presence. Some PPTs confirmed that parents took a more passive role than usual, and two caregivers did not participate in the session because they did not want to be video recorded.

### Ethical considerations

The study was approved by the Institutional Review Boards at Virginia Commonwealth University and University of Illinois at Chicago, and by the Norwegian Center for Research Data. The study held minimal risks for the participants. Both PPTs and parents gave their written consent of participation. We did not intervene with the usual care of patients and sought to minimize our disturbance of treatment sessions. In the written and verbal information, we emphasized that we wanted to explore the multiple ways of understanding and doing play in pediatric physical therapy and not do a critical evaluation of the PPTs’ intervention and performance. All data material and personal information is securely stored on servers for sensitive data at UiT and UIC. Personal and geographical information are excluded in transcripts of the material and participants are anonymous in our presentation of findings.

## Results

Our findings are based on the two main themes, each with three subthemes. We start with a rich presentation of findings, before we summarize and relate them to our theoretical framework in the discussion. View Supplementary material for all cited quotes.

### Understanding play and its role in physical therapy

This first theme elaborates the PPTs’ intuitive and at times elusive conception of play, and its necessary presence for making therapy meaningful for the child. At the same time, they highlighted their need to feel that their therapeutic approach is more than just play.

#### What play is and how it works

There was a general query among the PPTs regarding what play is, and they demonstrated a broadness of thinking when it comes to what they considered to be play activities. Social interactions, i.e., eye contact, vocalizations, and touch were explained as important early play skills. One PPT said: *“It could be just to touch something or touch your mom or look at your mom. This is all play; it does not mean you have to do a circus show.”*

Sensory experiences, exploration of toys, and experimenting with movement were all mentioned as play by the PPTs. As such, play is understood as a child’s exploratory and investigative behavior to learn about their bodies, objects, and surroundings. However, the PPTs sometimes found it difficult to untangle what it is that makes it playful, as in this quote about a boy who enjoys pushing a wheeled box of toys: *“He’s having fun for sure, but what makes it fun for him I certainly do not know. Because he does not seem to care at all about what’s in the box, so it’s not about moving stuff around. He is busy maneuvering the box, (…) but I cannot quite see that as play either. But it looks like he really enjoys maneuvering it, so maybe that is the play? Or is it the ability to move along with it? I do not know, I have no idea!”*

Similarly, another boy was excited to have a basket for transport attached to his walker, and the PPT wondered whether this can be understood as play: *“The basket enabled him to pick up and collect things and then gave a different meaning than just walking with a walking aid, you know like “I’m transporting something here.” (…) So it becomes a kind of play, or something similar to play at least.”* Later, this PPT discussed whether the engagement children often express while helping with tasks indicates that this is a type of play. She states: *“Well, it’s a joint project which they find meaningful, the same way that they find play to be meaningful. And it’s really valuable that they see this meaning in what they do.”*

To cope with this lack of clarity on what play is, several of the PPTs questioned if it is really necessary to define play. One PPT said: *“I think as long as it is something they want to keep on doing, I consider it to be play.”* But joyfulness is a key ingredient*: “As long as they have a smile on their face and want to keep the activity going, I do not see the need to define what it is that makes it play.”* The PPTs highlighted the connectivity between play and learning and emphasized children’s sense of meaning and mastery as important common factors: *“It’s how they learn, you know, because we learn when we do something that has significance and meaning for us. And what is significant for children? It’s to play, together with other children and with us.”*

The PPTs connected the dots between motor, cognitive, and social development, and considered play to be a means through which they as therapists can encompass all these areas of development during therapy. The understanding that motor skills and mobility drive development across domains also seemed to be well-established among the PPTs. Accordingly, they contextualized and evaluated the play activities during sessions in terms of how they can facilitate development in everyday life. This quote demonstrates: *“[With the push-cart] he can get up and move around, and you could see he was proudly transporting that teddy bear around. It was really nice that he mastered it so well. I think it’s important for his cognitive development, that he can actually move in standing, not always crawling, and (…) get a response up there.”*

The PPTs viewed play as a way to facilitate motor skill learning through varied mass practice in daily life. But at the same time, they reversed the idea; when the children make use of their newly acquired movement skills they also expand their play skills. One PPT explained: *“I think that is a sign of success, that he expands his play based on his learning of motor skills, and at the same time gets many repetitions and an automation of what we have practiced.”*

#### Play and therapy go together

The PPTs unanimously stated that play is the only way you get to do therapy with young children. It is the prime activity that children engage in and how you get things done: *“I do not know how you would get anything done if you are not playing! They’re just not gonna’ do it.”* This applies also to the very youngest infants and their way of playing: *“That’s really where I have to start to get anywhere, from when they are very, very young. (…) It’s that interaction and where the child’s attention is at, that’s where you have to find your way in.”*

Play is also viewed as the best way for children to find therapy meaningful: *“We have to engage and interact with them so that they experience it as meaningful. And if it’s not play then it’s hardly ever meaningful to them.”* Again, the connectivity between motor and cognition is highlighted, as exemplified by this quote: *“You have to get not just the body moving, but the brain has to choose to move. And why do we choose to move? We want to do something, want to touch something.”* Taking it further, it is not just about making therapy play-and meaningful; one PPT explained her overarching therapeutic goal: *“The most important thing is that these children learn to play, that we help them find ways to explore on their own.”*

This co-existence of play and therapy is initially explained as unproblematic among the PPTs, as this example demonstrates: *“I think it always works together, and I think I can always find a way to turn a play activity into some sort of therapeutic activity. It’s just that sometimes it takes a little more thought.”* Another PPT turned the argument around: *“It’s about figuring out how to turn an exercise into play. (…) Say I want to strengthen those quads, (…) how do I get a kid to want to do that? They do not want to do an exercise, they want to play. So, if I can get them to kick a ball that’s like long arc quads, or if I can get them to jump, or squat down to get a toy and get back up, then that exercise becomes a play activity that they enjoy doing.”*

The PPTs explained that play can build relationships during therapy, between themselves and the child or between the parent and child. Sometimes, allowing for free play is important to build trust. The PPTs also emphasized that they want the children to be active and interactive, so they can do therapy together with rather than on or to the child. One PPT summed it up: *“I may just let them play to get them to trust me, or to give them confidence that they can do something that they are really good at. (…) Or to help them regulate if they are having a hard time with something.”*

The PPTs explained that they want to be involved in the child’s play and do not encourage solitary play that the child excludes them from. But aside from this exception, they said that play is easily integrated into therapy: *“As long as it’s socially engaging play, any sort of relational play or contact play with another person, I do not think it can ever get in the way of [therapy].”*

#### I’m not there just to play

Although the PPTs said that they intuitively and easily integrate play into their sessions, they underscored that they also attend to their therapeutic purposes and goals. They value their role as promoters of development and see it as their task to keep pushing a child forward: *“I feel that my task is to be a spearhead of development (…). And with that comes this tension between having fun and accepting some striving. We’re supposed to move things forward, to achieve a goal (…). So, I do not want to be just another person who is nice and friendly* – *I can be nice and friendly* – *but I do not want to be just that.”*

Similarly, another PPT explained how play is always connected to a therapeutic goal: *“The play needs to be geared towards the goals that you are working on, and you have to figure out how to get the kids to play in a way that makes it therapeutic.”* By this, the PPTs make children endure more practice and challenge their motor performance, e.g., maintain new postures or try new tasks. But play can also function as a diversion for other therapeutic tasks: *“When it comes to her spasticity, how can we stretch without her resisting it? I try to find positions where she can play [while I stretch]. But then it’s perhaps not play, but more of a diversion.”*

One PPT explained how play can conceal therapeutic goals and thus increase a child’s motivation: *“My goal is to make the child think we are just playing and they are not doing anything extra, the goal is for them to not realize what I’m doing.”* Contrary to this, another PPT explained how she is willing to work through struggles to arrive at playfulness in the end: *“I want to find something that they like, I do not ask them to do a game they do not want to do. But if they are cognitively impaired, they might be agitated, because they do not know what to do (…). But I’m like ‘We’re gonna do it (…) I do not mind a little agitation’. And we work through it, and maybe at the end it is joyful.”*

### How to play in physical therapy

This second theme elaborates the PPTs’ explanations of how they manage the embedding of play in therapy. While joining in on the child’s play as part of therapy can sometimes flow smoothly, the PPTs highlight more the situations in which they feel that play and therapy disturb each other. In addition, they explain why it is difficult to play during therapy, and how they struggle to create play together with children whose interactional skills are different rather than typical.

#### Joining in on play

The PPTs explained that to create joint play, they prepare the therapeutic space before sessions. Some opt for a structured set-up with specific toys and equipment to gear the child toward targeted motor and play activities. Others provide a broader selection of play and environment opportunities: *“That’s my strategy for this age, I lay out the stuff and then allow them to choose what they might want to do.”* In the quest to join play with a child, an important first step for the PPTs is to patiently spend time and discover the child’s play interests for the day. Next, they invite themselves in as a play partner and adapt their behavior to develop an interactive play activity. One PPT explained: *“Often I kind of give up on my project for a while and join the play. And then I wait and see, still with my agenda in the back of my mind, and then I’m often able to change the play to something that is more up my alley. But I have to give it time. I cannot arrive and say ‘Now we have half an hour’ and do this and that.”*

Similarly, another PPT explained: *“For the most part, I’m following the child’s lead and try to be creative and come up with ways to work with the child and still attain some therapeutic goals.”* This gradual approach makes the PPTs discover new play opportunities, e.g., a child manipulates objects in new ways or does role play for the first time. But they also have the therapeutic goal in mind and look out for new motor capabilities that may emerge through play. It is only one PPT that admitted that she could sometimes immerse herself completely in play: *“I’ve caught myself joking about it a couple of times, that I realize that I’m not working towards a goal, it’s not therapeutic anymore.”*

Having the parents join play was also important to the PPTs and they explained that they tune themselves in on how the parents typically play with their children and adapt their therapeutic approach accordingly. They also exchange information about a child’s play preferences and possibilities in their dialogue with parents and encourage parent–child play interactions during sessions. By this, they can guide parents in finding new ways to play with their child and pick up on play ideas that they can expand on during therapy sessions.

#### When play and therapy get in each other’s way

In stark contrast to the overall view that play and therapy go together, the PPTs brought up tensions between play and therapy. One PT explained how a priority of play disturbs her in her work: *“It has to be playful, but (…) sometimes I feel that it all turns into nonsense. (…) I know that I have to work through play, but how do I get to what I want, the movement quality and such?”* Another PPT drew attention to how play dissolved due to an eagerness to do movement therapy: *“I got excited on his behalf you know, and then play disappeared and there was just movement, without much play.”* Contrary though, the therapeutic tasks of guiding movements can sometimes be important facilitators of play, as explained by one PPT while watching a play sequence: *“What is my role, should I be the one who runs the [play] interaction, or the one who tries to do something with arms and legs? The interaction is left to the assistant. I could probably have organized things so that he was facing me and interacting with me. But I was happy as it was, my task here was to create good positions for him.”* Finally, one PPT who also acknowledges this tension between play and therapy took a clear stand to prioritize play: *“It should be on the child’s initiative, you have to figure out what makes the child want to put in the effort, a joint project that is enjoyable, playful, and motivating. (…) I think we as therapists should be better at using movement pleasure and play rather than focusing on the correct movement patterns.”*

The PPTs highlighted the predicament that arises when a child does not welcome motor challenges as part of play, as this quote demonstrates: *“The child will choose what he’s confident in and what he already knows. So there is this balancing act, where you want to challenge him* – *in a nice way* – *through play. And every now and then I’ll lose someone there because they do not want to get into stuff that they feel is difficult.”* The PPTs also explained that some children are quick to complain if they feel that you intrude on their play. Or they will simply not play along with what they perceive as a play disturbance: *“I look at where I can build in a bit more of a challenge. But then the child is not interested at all [in my suggestions], that’s very often the case. So, my project collides with that of the child.”*

One PPT referred to using toys as bait and explained that this is something to be cautious about: *“There is this risk that toys become baits. We have to let them grasp. Not just use it as bait and then take it away, use it as bait again and then take it away again. Because then they will resign, and some children resign very fast. So I try to be aware that when I offer a toy, the child is allowed to investigate and study it, turn it around, I think that’s important.”* Similarly, one PPT explained how she thinks the therapist’s agenda and leading of the play can be disturbing even for the youngest children: *“I use toys to elicit a certain activity. But maybe it’s not really play, because I have an agenda, and I think that disturbs the genuineness of play. Just like when you intervene in role-play [with older children], an adult mistakenly steps in and directs* – *consciously or unconsciously* – *how to play. I think that can probably happen with infants as well.”* Delving deeper into this issue, the same PPT questioned how play can be upheld and to what extent goals can really be shared and between the therapist and child: *“The play often has a goal within itself, let us say we want to build a tower. But then I add some external goal, that’s when it starts to get troublesome. (…) It’s a joint project, where I have my intentions. But I do think it’s important that we create something joyful together, that the child feels ownership of and wants to keep on doing. But we are not really equal, so can we still call it play?”*

A more radical challenge in the combining of play and therapy is when a child’s play counteracts the therapeutic goal. E.g., in one of the observed sessions, the PPT was working with an infant and targeting elbow support in a semi-kneeling position with elbow support on a wedge, facing a mirror. The infant eagerly reached to touch his reflection in the mirror, and thus collapsed from his elbow support and down into prone. In the next attempt, the PT thus restrained his arms from reaching toward the mirror, to maintain his position for a longer duration of time. The PPT reflected on this situation during the interview: *“The problem is that when he tries to [reach for the mirror] he loses his position. I would gladly let him do it if he had the skill, if he was able to use a single elbow support, then I could have let him reach. But it was too difficult.”*

#### Play is hard

Although play is a natural way of doing therapy with children, the PPTs admitted that it can be hard to create play and stay playful during sessions. One PPT explained how it gets her out of her comfort zone: *“You just have to roll with it (…). I think the fact that it’s so unstructured and so unpredictable is what makes it entertaining, and keeps you challenged. (…) It was hard when I started out doing it that way, but the benefit is that you are being challenged more, you are thinking outside the box.”* Confirming this unpredictability, another PPT listed up a range of factors that are involved in making play flow easily or causing it to not flow at all: *“Sometimes it just does not work. and then I try and figure out why. Is it our chemistry? Is it about me? Is the child out of shape, or did not want to, or was it just the wrong day? Or is this an insecure child? It’s not always easy to analyze.”*

Experience in the field has for the most part helped the PPTs in developing a more flexible approach, as one PPT says: *“It was much more difficult when I was a fresh graduate. Now I have a larger repertoire to play with, so I can just change. Change my role or focus in play or type of play, to get where I want.”* But with experience, play can also become a repetitive routine of what you typically do with a child, or across sessions with different children, as another PPT said: *“I’d like to become more aware of how I use play. I think I end up doing a lot of the same thing, it quickly turns into a routine.”*

A common experience among the PPTs was that it is challenging to approach children who present with stereotyped play or who are difficult to engage in novelty experiences that may help drive development forward. Another example is children apraxia tendencies and thus struggle to figure out how to play. One PPT explained: *“When a kid is not able to expand on their play skills. [Because] I do feel that it is ok to meet a kid wherever they are developmentally. But when they cannot expand upon that, to be able to really progress their gross motor skills, it becomes challenging.”* Another PPT pointed at the lack of interest to move as a personality trait that is difficult to work around: *“The kids who are not motivated to move are the hardest kids for me to work with. Because there is nothing, no play entices them to do what I want them to do.”* Similarly, the PPTs found it difficult to establish and share play with children whose social interaction skills are delayed or atypical, or those children who prefer to be an observer. They highlighted the importance of shared experiences in play: *“I find it most challenging with the children with whom it is difficult to interact. Or those children where it’s difficult to find a type of play that they can master. (…) [If they do not get] that interaction and development in togetherness with others, I think it’s a big loss. [They need] to interact with others, see and explore and learn [from each other].”*

When such interactional play difficulties occur consistently over time with a child, the PPTs start to question the cause of it and look for autism spectrum signs and symptoms. Other cognitive deficits and delays are also mentioned as barriers of play and social interaction. Along these lines, the PPTs consider cognitive capacity to be crucial to a child’s drive to play. This quote relates to a session with a 4-month-old child with Down syndrome: *“I do not see him make any good eye contact. (…), and there is little response in the arms. (…) He’s making it quite clear that there are several things he’s not ready for yet. And I try to take that in, that the play interest is just not there.”* The challenges of playing with children with severe disabilities and delays across motor and cognitive domains of development are also underscored by the PPTs, one of them said: *“The most challenging are children who are functioning at a very low level. Trying to have them find ways to access play activities can be very challenging. The cognitive piece of figuring out what’s going to motivate them, and then the motor piece of figuring out how they can access whatever it is we find [to play with].”*

The PPTs view the children’s motor skills as one piece in the big puzzle of abilities that all develop through and during play. When a child struggles with play for any of the above-mentioned reasons, the PPTs will consequently struggle with doing therapy with that child. The child’s engagement and motivation are difficult to tease out, and it is hard to find ways to work on specific skills. One PPT highlighted that it is important to continue the effort because children can suddenly “wake up” and enter a cascade of developmental improvements once they start to experience some level of mastery. This most typically occurred with children who recover from temporary medical conditions. Another PPT pointed at such a “wake up” as the most significant event of the observed session: *“I think that was the biggest thing [today], he was finding things motivating enough to want to try to do something. That was big! And new! Not just watching the world around him, but really engaging with toys and being independently motivated to play.”*

## Discussion

Summing up our findings, the PPTs’ confirm the elusive and diverse nature of play that is already described in the literature (Lifter et al., 2011; Yogman et al., 2018; Henricks, 2015a; Henricks, 2008); they intuitively know what play is but struggle to define the phenomenon. Nonetheless, aligning with the definition of play provided by Fiss et al. (2023), the PPTs recognize play as meaningful activities that children enjoy and want to keep on doing. They also highlight play as social and interactive learning activities. From our enactive stance (Varela et al., 2016; Froese and Di Paolo, 2011; Di Paolo et al., 2010), we interpret that the PPTs acknowledge play as fundamental to children’s sense-making and learning across domains and as a vital component of therapy. When play and therapy can emerge together and co-exist, it fosters engagement, builds trust in the PPT-child relationship and becomes a vehicle for children’s skill development and sense of mastery.

While the PPTs have an overall view that play and therapy can co-exist and intertwine, they often experience tensions between their therapeutic agenda and the genuineness of play during therapy sessions. In their sense-making and clinical reasoning, they strive to make play therapeutic one way or another, and frustration arises if they perceive activities as just play without therapeutic value. The PPTs find it particularly taxing to join sense-making and play with children who present with stereotypical behaviors or limited social interaction and play skills. Play does not emerge easily between them, and they find it difficult to identify and incorporate new therapeutic elements into play.

### Making play a therapeutic agenda

The PPTs’ view of play as a foundation for learning across domains signals that they understand children’s development holistically and recognize how cognitive, motor and social skills co-exist and co-influence each other. The PPTs recognize play as the context and activity through which these developments happen, as exemplified by the quote about the boy with the teddy bear in the push-cart. This aligns with the enactive theoretical framework and the idea that children learn to enact their world through play.

In their work with children aged 0–3, the PPTs acknowledge that play is the way to get things done; it is play that engages and makes children attend and find meaning in the therapeutic activities. This tells us that the PPTs view play as a sense-making process for the child and that they strive to connect their therapeutic purposes to play activities that are meaningful and motivating for the child. The PPT who sometimes abandons her project and then with time finds her “therapeutic alley” within the child’s play is a key example. The enactive term of emergence can elucidate her attitude; she facilitates mutuality between her and the child, with play as the medium in which the child can show his or her intentions and engagements, and through which new therapeutic opportunities can emerge. This mutuality comes about because she accepts play to be the first and foremost agenda and is willing to set her therapeutic agenda to the side for the time being.

This example is however an exception from the more typical tensions between therapy and play that the PPTs verbalize as “I’m not there just to play,” or the sensation that play interferes with their ability to be specific enough. In addition, there are occasions when the child’s play is disturbed or disappears because of the PPT’s therapeutic agenda. The richness of such examples in the material indicates that mismatches between play and therapy are frequently occurring events and a challenge that is difficult to overcome. We propose that these mismatches stem from a fundamental misalignment of intentions between the PPTs and children. Although the PPTs acknowledge play as a way of learning for the child, they do not enter the interaction with a genuine intention to play. Their concealed, therapeutic agenda is still front row in their mind and play serves as a medium for therapy rather than an activity that is valued for its own sake. As a counter example we consider the statement of the PPT who wants the child to feel that they are “just playing” and not “realize what I’m doing.” Still attending to her therapeutic goals, the PPT is not a content player – or therapist – until the child perceives the activity as genuine play. An enactive interpretation is that this PPT values the child’s experience of play and that an intention to play is an integral part of her therapeutic approach from the outset.

Expanding on this, a genuine play intention during therapy does not imply that PPTs should be content with “just playing.” Therapeutic play is a matter of finding, integrating, and maintaining therapy during play. Recognizing and responding to a child’s play intentions, initiatives, engagements, and movement explorations within the play supports a therapeutic approach that becomes uniquely tailored for each child. This requires making play the starting point and discovering how it can improve therapy; how it challenges you to “think outside the box” as phrased by one PPT. Connecting to enactivism, it requires a sense-making process that welcomes the child as a mutual decision-maker in the pursuing of significant events and actions during therapeutic play. In addition, it requires a PPT’s ability to derail from standardized plans and approaches, be flexible, and manage unpredictable turns of events.

However, our findings also indicate that there are some situations in which “just play” can be the therapeutic agenda. If the relationship between a PPT and a child needs to be established or improved, play can be a necessary investment that enables future therapeutic opportunities to evolve. In enactive terms, genuinely engaging in a child’s play supports the child’s agency and allows the PPT to explore the child’s sense-making and understand what is significant to the child in that play.

### Making therapeutic play easy

The many challenges of therapeutic play described by the PPTs underscore the frailty of PPT-child interactions and confirm that it is often difficult to share play intentions when they enter play with different intentions from the start. One fundamental challenge is the contrast between the structured, goal-directed interventions that PPTs are trained at (Bexelius et al., 2018; Pritchard-Wiart and Phelan, 2018), as opposed to the spontaneity and freedom to explore that play entails (Henricks, 2015b). An example is the PPT who feels that “it all turns into nonsense” and wants to be more specific than a play approach allows for. We suggest that commitment to the understanding that play is learning for the child can ameliorate this challenge. A feeling of sense or nonsense then is not about what the PTT wants to achieve, it is about supporting the child in how he or she wants to play and learn. Accepting this standpoint makes it easier to set structures and plans aside and promote explorative movement and play as a therapeutic approach.

Creativity in this merging of play and therapy is an important therapeutic skill to develop and apply. In our material, one PPT says that experience has given her “a larger repertoire to play with,” while another PPT says that she ends up doing “a lot of the same thing.” Enactively speaking, a PPT’s easily available adaptations may be a result of a more mutually emerging and flexible therapeutic play approach, as opposed to a routine based PPT who is less flexible in regulating her intentions and actions to those of the child. Creativity then, is a matter of cultivating a therapeutic approach that evolves through play, rather than simply framing therapy in a playful context or disguising therapy as play.

Similarly, the PPTs describe that their therapeutic effort of building motor challenges into the child’s play often collides with the child’s play project. In enactive terms, this can be understood as a failure to coordinate their therapeutic intentions with the child’s play intentions (Håkstad et al., 2017). As a result, both play and therapy come to a halt. However, these interruptions should not be viewed solely as setbacks. During interactions, interruptions are inevitable and can become significant sense-making events that the therapist and child can explore and that can develop into new paths of understanding and interaction. For example, a new sense-making might be that the PPT considers more carefully the type and extent of motor challenges the child is willing to accept. This is a participatory sense-making in which the child and PPT explore and balance the gap between the child’s skills and the challenges provided. The individual child’s willingness to struggle and the level of success needed to uphold the child’s engagement are important factors to take into consideration. Notably, both play and learning can thrive on teasing and struggles (Reddy, 2015), so finding ways for a child to enjoy effortful challenges or finding a closer match between the child’s current abilities and smaller gradient of challenge during play can be an important therapeutic ingredient. Another challenge portrayed in our findings is that play becomes difficult when the PPTs compartmentalize developmental domains or focus narrowly on the motor domain, e.g., the PPT who got excited about biomechanics and handling techniques and so “play disappeared and there was just movement.” Such compartmentalization may stem from a learning about development in which the different domains are presented in isolation and with insufficient explanations of their interdependence. Other statements indicate a more holistic approach; the PPTs want to engage the children in meaningful interactions that make them want to move and put in an effort during therapy. Literature and education that argues for a more holistic understanding of children’s development, acknowledging the child as a self-exploring agent and interactional partner (Lobo et al., 2013; Sørvoll et al., 2023) can support PPTs in such clinical reasoning and approach. In enactive terms, this clinical reasoning entails a mutual sense-making process in which the child’s agency, intentions, and play engagements are important drivers for movement actions. When PPTs integrate such sense-making with their knowledge of biomechanics, movement analysis, and strategies to facilitate motor improvements, they can more likely deliver therapy that is interactive, meaningful and playful.

The challenge the PPTs encounter with children who present with stereotypical behaviors or limited social interaction and play skills is an important issue to unpack. Their descriptions underscore the PPTs’ belief that play and learning are intrinsically linked; without play, a child’s capacity to learn new things is hampered. Furthermore, it highlights that a child’s ability to express and share intentions is fundamental to developing mutuality in the interactions between the PPT and child. But these expressions of intentions also rely on the PPT’s ability to perceive and interpret the child’s signals and respond to them. With children who have limited interactional capacities, bodily communication and engaging in activities together are crucial for participatory sense-making, enabling the PPT and child to co-create what their goals and achievements. When a PPT struggles to understand a child in such interactions, their participatory sense-making breaks down and leaves the PPT with a perception of the child as someone who is difficult to communicate and work with. Within the enactive framework, we propose that PPTs need to strive to recognize and understand how these children express what matters to them as agents and how they do their sense-making (Håkstad et al., 2017; Fantasia et al., 2014; Fuchs and De Jaegher, 2009; De Jaegher, 2013). Identifying a child’s interaction initiatives and skills within what may appear as limitations or stereotypical behaviors can make it easier for PPTs to work with the child. This mandates that the PPT embraces creativity and explores interactions that are less conventional for the PPT, but familiar or typical for the child. Dialogue with the caregivers and learning about their ways of interacting with their child can be a valuable source of information in this regard. By exploring these less familiar forms of social interaction, PPTs can uncover new opportunities to connect and participate in the child’s sense-making that in turn can serve as gateway for playful therapy.

The PPTs’ description of children who are perceived as difficult to play with also prompts reflection on the capabilities of the PPTs themselves and how they understand their role and responsibilities during play interactions with these children. A depiction of children as lacking the drive to play or not able to expand on their play can to some extent also reflect a shortcoming in the PPT’s ability to create engaging and dynamic play interactions with this child. If play is to be co-created and emerging through interactions, then both parties share the responsibility for its generation and progress. Reframing the idea of a child who is difficult to play with through introspective questioning of the reasons behind this perception is helpful. This is exemplified in our material by the PPT who considers a range of factors relating to her own actions, the child’s perception and comfort in the situation, and their interactional chemistry. In enactive terms, this is an acknowledgment of how the therapeutic intentions, the child’s intentions and concerns, and the dynamics of their emerging interactions together shape their abilities to play and do therapy together. We suggest that adopting such a reflective stance on one’s role and accountability as an interactional partner is a crucial step in clinical reasoning-in-interaction (Øberg et al., 2015) that can help understand and resolve interactive play challenges with a child. By exploring alterations and expanding their therapeutic and interactional approaches, PPTs may unlock new possibilities for play engagement and capabilities.

### Playing with play in therapy

Based on these discussions, we want to highlight key implications for PPTs’ understanding and management of embedding play in physical therapy. First, we suggest that clinicians playfully explore the possibilities and potentials of play in clinical practice. Understanding how to make play the starting point of therapy is crucial. As one of the PPTs aptly noted, it is in “that interaction and where the child’s attention is at” that you must find your way in. Starting from play allows therapy to evolve through a shared sense-making process, with both the therapist and the child contributing to the emerging movements, actions, and interactions. This collaborative dynamic can then turn into an individually tailored intervention strategy, uniquely crafted through the interplay between the PPT and child. Making play the starting point entails that clinicians must trust the intrinsic value and learning potential of play (Yogman et al., 2018), allow play to emerge and lead the therapeutic interactions, and grasp the therapeutic opportunities that evolve through this play.

Next, we suggest that play can infuse most aspects of therapy, including the integration of therapeutic challenges; a child’s striving and overcoming of resistance can become an ingredient that makes play thrive, as long as it is not forced. Even more, activities that may initially feel like work can transition into play as a child’s motor skills improve and tasks become more manageable. We invite PPTs to experiment with these balancing acts and boundaries of play and let the emerging interactions with the child co-determine how therapy unfolds. Respect for the child’s autonomy, attention to the child’s play intentions and experiences, and repairs of potential interactional mismatches are crucial in this process.

Finally, we suggest that PPTs reflect upon their use of play and find ways to engage children in play that is genuinely shared and mutual, and that upholds the child’s autonomy. By this, the tensions that naturally arise may be perceived as less challenging for both the PPT and the child, because they are recognized and resolved through dynamic and flexible participatory sense-making (Fuchs and De Jaegher, 2009). We conclude with this statement from one of the PPTs: “The most important thing is that these children learn to play, that we help them find ways to explore on their own.” This highlights learning to play to learn as a way to help children develop motor skills, gain a sense of mastery, and enjoy the freedom to explore their bodily possibilities as they venture beyond what they already know.

### Study limitations

This study investigates Western settings of play within physical therapy, and we do not have data on how play is understood and managed in therapeutic settings in other cultures. Understanding the role of play in diverse cultural settings could provide a more comprehensive view of its therapeutic potentials and applications. The age range of children 0–3 years limits the relevance of this study when it comes to the integration of play in physical therapy for older children. There is also an underrepresentation of children with more severe disabilities. This gap highlights the need for research that delves into the therapeutic play experiences in interactions with these children. While our study emphasizes the importance of play in therapy, we do not assert that all therapeutic activities must be playful. Some aspects of therapy may simply need to be tolerable, but this is beyond the scope of this study.

The role of parent and caregiver involvement in therapeutic play was not the focus in this paper. However, given the pivotal role that parents and caregivers have in children’s lives, future research should examine how PPTs engage parents in therapeutic play and how such involvement provides mutual learning opportunities and impacts therapeutic outcomes. Another area that requires further exploration is the involvement of other healthcare professionals in therapeutic play and the similarities and differences across professions.

## Data Availability

The datasets presented in this article are not readily available because the informed consents restrict such sharing. Data summaries are provided as Supplementary material. Requests to access the datasets should be directed to ragnhild.hakstad@uit.no.
