# Supplementary material for: Learning to play to learn in pediatric physical therapy
Source: Front Psychol. 2025 Jan 6;15:1467323. doi: 10.3389/fpsyg.2024.1467323 (PMC11743683; doi:10.3389/fpsyg.2024.1467323)
Supplement: Supplementary file 1 [file Data_Sheet_1.pdf]

Supplementary material: Learning to play to learn in pediatric physical therapy

Themes, codes and meaning units

Understanding play and its role in physical therapy – codes:

What play is and how it works:

Bodily experimental play
Bringing stuff creates meaning
Create playful explorative learning opportunities
Child enjoys pushing things around
Child's play provides repetition and variation
Children enjoy helping out as part of play
Explore and experience through play
Free and facilitated play
Important that the child learns to play
Interaction is play
Is helping out play?
Is pushing things play?
Mobility as a driving force of development
Motor-social-cognition interplay
Play as a mode of engagement with the child
Play encompasses all areas of development
Play engagement improves motor performance
Play is development, learning, relation, mastery, participation
Play is interaction
Play is learning and meaning for the child
Play regulates and engages the child
Must play to provide quality intervention
Play builds self-esteem and confidence
Play is a child's work
Play is unstructured and unpredictable

Play and therapy go together:

Child tolerates play interruptions
Children should perceive therapy as play
Combining movement and play
Engage the child and build therapy from there
Engage the child and build therapy from there
Expanding the child's play repertoire
Help the child discover play
Merging of play and therapy
Motor challenges flow naturally in play
Must play to get things done
Play comes naturally
Play is a must
Play is how you make your way
Play is the only way to do therapy
PPT's role is to create bodily opportunities for play

Reaching therapeutic goals through play
Relational play never gets in the way of therapy
Support child's motor background of play
Therapy connects play to goal

I'm not there just to play:

Anything can be adapted to play
Can work through agitations
Challenging to make play therapeutic
Creative to attain therapeutic goals
Expanding play to progress motor skills
Finding a balance between striving and play
Hard but rewarding to turn exercise into play
Have to play – but is it play?
Join play and then get in on the PPT track
Play sometimes means less specificity
Pushing the child is the PPT role
Therapy as a balance of fun and striving
Turning exercise into play
Turning play into exercise
Using play as distraction

How to play in physical therapy – codes:

Joining in on play:

Adapting to the child's play
Allow child to be the leader
Child arranges play
Child co-creates play environment
Child connects to PPT's play ideas
Child engaged in familiar play
Child enjoys structured play
Child initiates mutual play
Child is easy to engage in play
Child-PPT play partnership
Easier to play with more experience as PPT
Easier when the child has a drive to play
Get interactive play going
Mutual play facilitates motor performance
Mutuality takes time to establish
Play disturbances are quickly repaired
PPT wants to be part of play
PPT-child play routine
PPT-mom-child triadic play
Sometimes play takes over
Think outside the box

When play and therapy get in each other's way:

Ambivalent to using play in therapy
Can't play and do therapy simultaneously
Child resists PPT's arrangement of play

Child switches between protest and play
Child’s play initiative is disturbing PPT’s intention
Children want to play not exercise
Exercise rather than play
Play sometimes means less specificity
PPT and child projects colliding Play as a means rather than goal
PPT interrupts child’s play
The PPT’s agenda can disturb play
Things often do not go according to plan
Using toy as bait can discourage the child

Play is hard:

A fine line between agitation and mastery
Children with autism and rigid play
Monotonous play
Children who do not know how to play
Challenging to find ways to play
Child does not know how to play
Child is difficult to engage in play
Child is finally starting to play
Child shows little social interaction
Child wants to explore in solitude
Children who do not play
Children with autism and rigid play
Difficult when interaction is lacking
Difficult when motivation is lacking
Difficult with children with low motor functioning
Hard to detangle what goes wrong
No drive to play
Searching for the child’s play interest
Social engagement with the child has been difficult
Trying to catch the child’s interest

Understanding play and its role in physical therapy – meaning units

What play is and how it works

I figure as long as they want to keep at it, it's all play to me.

I think it's important that we manage to create something joyful together, that the child feels ownership of and wants to keep on doing.

It could be just to touch something or touch your mom or look at your mom. This is all play; it doesn't mean you have to do a circus show.

Yeah, we don't always understand, because what's play for me isn't always play for you, you know, and that's where kids differ in what they consider play. But I figure as long as they have a smile on their face and want to keep the activity going, I don't see the need to define what it is that makes it play.

Why is it fun to go around pushing and shoving then? Why is that play? Or is he using it to move himself? He's having fun for sure. But what makes it fun for him, I certainly don't know. Some might think they're just rolling it along, but he doesn’t seem to care at all about what's in that box. For some, it might be something, moving something from one place to another, right? But that's not where his focus is. It's on maneuvering it. But I'm thinking, is it the act of moving around walking, just that he has something to hold onto in a way? That in itself is play? It's kind of like, how do you define play then? It sure looks like he's really enjoying maneuvering that box. So that's kind of what the play is, right? Or whether it's a game or what is it? Or is it movement, managing to get somewhere, no I don't know, I have no idea!

I really want his play to be as varied as possible. He shouldn't be dependent on me or others to carry out his play, or to be able to join other kids in play. He should be able to interact well with other children in the group, in natural play that isn't directed by adults. So, my goal is for him to be able to do what he did today on his own, without me.

They should have free play as well. They can have facilitated and free play, right? Like why can't you do both?

I really felt it clicked with that walking aid. Adding a basket to it meant he could pick up things and collect things, and that gave it a different meaning than just walking with a walking aid, because I'm carrying something here. Like a kind of play, or something similar to play at least. I get it, I also like to carry things when I'm walking and doing stuff. It's really not that strange. I rarely consider walking as play; it's a means to fetch something or do something else. So, it's about trying to make it so that we're not just walking here to practice walking.

Feeding fish might not be play in the sense of creativity, exploration, and building and stuff. But at the same time, it's something that motivates, that's exciting, a joint project, something they get involved in and look at, and can sing songs about, right. It's definitely a joint project, something they find meaningful. And I also find that they find the play very meaningful, so it's something that has a really good value, the fact that they see the meaning in it. So maybe it's not so important whether we call it play or a joint project or whatever definition we use.

He's up standing and can move around wherever he wants at that height. So, I put some things in the wagon, like transporting a teddy bear, he was quite proud so it was really nice that he mastered it so well. I think the sense of achievement has a lot to say for his cognitive development, that he can actually get up and move in standing , not always crawling. And if the grandparents are there, he can go over to them and then get a response back up there.

It’s how they learn, you know, because we learn when we do something that has significance and meaning for us. And what is significant for children? It’s to play, together with other children and with us.

I think it is super important. I don't know how you would get anything done if you're not playing! They're not gonna do it. And you can't ignore the language and the cognitive part of it. And especially in EI you are maybe

one of the first people, so you're showing the parents how to play with their child at this age. These are toys that are appropriate, even just looking at your face and talking is appropriate. And then with the older kids, again I don't know how you would do anything. It's a good opportunity to work on multiple things. Especially for kids that are not typically developing, I feel that it's a bigger deal for those kids, when the parents don't get those reactions back, the eye contact or the vocalizations, so then making sure that they are still interacting, and that we are still interacting in those ways with the kid and not just sitting there, doing therapy on them, but they are active, you know.

I think that play is an important part of children's development. And they learn through play. And playing with the children, I think, builds a good relationship and contact, and maybe you can also give them some good experiences of mastering and participating.

I think that is a sign of success, that he expands his play based on his learning of motor skills, and at the same time gets many repetitions and an automation of what we have practiced.

Play and therapy go together

I don't know how you would get anything done if you're not playing! They're just not gonna' do it.

So I try to be mindful of that, when I offer a toy, that they are allowed to investigate and study and turn it around, that's important. But is it play? It is, when it's the child's exploration and curiosity about what this is, and how can I use it. The most important thing is that they learn to play, that we provide support so the child can explore on their own.

If I can create a good situation where he gets to use his skills together with other children, or with the assistant, with mom or parents, then that's more important than exactly what happens there and then with me. Because I might only have once, an hour, or at most two hours a week available. So, this thing with parental guidance, guidance of an assistant who might be with him a full daycare day, that's really where the opportunity for influence lies. I think that is a sign of success, that he expands his play based on his learning of motor skills, and at the same time gets many repetitions and an automation of what we have practiced.

Often, we start with what's available in the department, where the children are playing and what they are doing. Then we try to see how we can stimulate him to play on the different apparatuses, and how can we do the same outside? How can we use the play equipment in such a way that he doesn't think of it as training, but as fun exploration and play. And that we get many repetitions and an automation of the skill.

You have to get not just the body moving, but the brain has to choose to move. And why do we choose to move? We want to do something, want to touch something.

Yes, it's a goal to work on the child's terms. Understanding what the child wants to do, and being able to facilitate what is appropriate to elevate it to a new skill level so that development can continue. I think it's important that the child achieves motor skills, or that it's facilitated as well as possible so that the child can master their interests.

We can't get anything done if we don't play with the kids. We have to engage and interact with them so that they experience it as meaningful. And if it's not play then it's hardly ever meaningful to them. Using food and iPads is a bad idea, so that leaves us with play, so we're doing that all the time.

Play is really the be-all and end-all. That's really where I have to start to get anywhere, from when they're very, very young. It's that interaction and where the child's attention is at, that's where you have to find your way in.

That's what I'm a bit wary of, in the play, not scared, but I feel that my task is to be, as a therapist, a spearhead, the one who takes him forward. And in that, there's a kind of tension between just having fun and struggling a bit. We're supposed to take him forward, we're supposed to have a goal in mind. We need to be aware that it doesn't just go upwards and upwards, but that it often goes in steps, an automation process of a

new skill, but still, we need to have a way forward. So I'm not supposed to be just another nice and kind person. I can well be nice and kind, but I'm not supposed to be just nice and kind.

I feel like that my primary mode of engaging with the child is with play. So I’m always using play, to motivate, to relate and engage with them.

I think it always works together, I think I can always find a way to turn something, some play activity into some sort of therapeutic activity. Sometimes it takes a little more thought, but usually we can figure out some way to make it therapeutic.

I was pretty satisfied with what he was doing. It's all about repetition you know, he was doing it on his own, he's getting lots of repetitions with this being away and coming back.
I think I would have been more frustrated or feel that I needed to do something if he was just being still and not moving as much as he is.

When I first started working in peds I went to a bunch of lectures, which were all about play, integration of movement, cognitive therapy or development, language development, social development. So, mobility is really important for other areas of development, no matter what that mobility looks like. Some mobility is better than no mobility. So, I've always kind of had that important piece in my head of how it's such a driving factor.

It depends on the play, as long as it's engaging play, socially engaging. If you count play as them watching something that's not necessarily beneficial per se, but any sort of relational play, or contact play with another person, I don't think it can ever get in the way of it [therapy].

That's kind of why i got into pediatrics, i didn't like all the boring structure, just get to play all day.

Sometimes you can achieve a fantastic spontaneous play and exploration. Just by providing a little support, and then suddenly something can happen. It often happens quite unexpectedly. Or very often it happens spontaneously and unexpectedly, that suddenly, you can see in a way that they are learning. That something worked, right? If you manage to provide support in the right place at the right time, things can start to click. And then the parents come back the next time and say that now he has started to do it, that there has been a development.

I don't think I could work with children if I didn't have play as part of it. I don't know how I would manage to get there, right. And at the same time, I wonder, what is the play here, in a way. I'm just thinking out loud, because I can't imagine treating children without using toys or play. Because that's the way they explore and learn. At the same time, I wonder, what I do as a therapist, is it play?

I feel like with kids that is their goal, in their development. They could care less about an exercise per se, but they want to play. And it's about trying to figure out how to turn an exercise into play. It's sometimes the most challenging aspect of what we do. But it can also be the most rewarding at the same time. So, it's easy to say I want to strengthen my quads. So, to do that I need to kick my leg out or I need to do resistance against my quads to get my quads strong. We all know that, but then it is how do I take longer squats, or whatever it is I want to do for strengthening the quads, how do I get a kid to want to do that. They don't want to do an exercise, but they want to play, so ok if I can get them to kick a ball that's like long arc quads, or if I can get them to want to jump or squat down and get a toy and get back up, then I’m taking that exercise, and it becomes a play activity that they enjoy doing. Q: Is it sometimes the other way around, that you have to turn their play into exercise? A: yes, that is true! That becomes even more complicated. It's weird to do it the way I mentioned first. So sometimes their play does not include therapeutic activities, especially like sometimes they want to play sitting, well I want them up and moving, and it's hard to take that activity and then decide how do I make that therapeutic. That is super challenging sometimes. They may be like oh I love to read books, and they want to sit and read books. Well, that's great, but it is not getting you up and out of the floor to walk over there. So, trying to figure out how to make that therapeutic.

I may just let them play to get them to trust me, or to give them confidence that they can do something that they're really good at. (...) Or to help them regulate if they're having a hard time with something.

I’m not there just to play

We've had quite a success with something we call the 5-year group. We have an intensive program. It's mainly kids who have been referred because they are a bit immature motorically, they have low self-confidence. And there we've received great feedback, like wow, now he's all over the play area. That's actually the main goal, to give them a bit of confidence that I can do this, and through repetitions, they notice that okay, I can handle this, and then they use it afterwards. And it's playful, there are obstacle courses, and then we have some activities based on the challenges the kids have.

He has major challenges with handling and mastering his body, so I think that creates insecurity. So, the way in with him was to sit and stand. And his stability has gotten much better than it was. I think about the fact that he gets experiences, gets to try, gets to feel, gets opportunities, gets starting positions where they succeed, while it is enjoyable and something he sees the benefit of, because we play and because there is interaction and a common focus area, or a game.

I didn't have any plan for what I was going to do with that ball. So, it was a bit about how much should I let him control, and how much should I try to work him into what I had in mind? But then I thought okay, I'll take down the ball, I can try to get him to stand up after a bit of an unstable thing. But then I try to get him to stand up after it, trying to get him to move a bit, roll on the ball, but it might be too demanding for him. It's about taking in what captures the interest, and then using it for something sensible, right, it's about adjustment. Then I see that I can't get him to stand up.

I see that it's often what I do, I try to capture interest in something, and then I try to move it where I want him to go. Sometimes it works and sometimes he moves on to other things because I can't keep his interest.

It's about trying to create a game within that area that also has some therapy in it, you know, that it brings an increased level of function.

My goal is to make your child think we're just playing and they're not doing anything extra, the goal is for them to not realize what I'm doing.

I'm definitely there pushing a bit every time, I am. And I think that might be my job, I really think so. You know, as a therapist, right, I come from a training background, with sports education, so I feel we need to practice what's difficult, if we're going to make it. It can't just be play, at least that's how I see it. I mean, it shouldn't be that he starts crying when he sees me, that's not what we want. It should be okay when I show up. At the same time, I think, the mom was tired, felt like he wasn't achieving anything, then we really need to push him, get him to utilize his resources as well as he can.

The goal has been walking and strengthening, so we've been going between that chair and that box, that's been the main thing we've been doing. And then try to get her to cruise around and occasionally pick things up. Normally mom is at one end and I'm at the other. And then one of us will build a tower and then encourage her to come over, "hope someone doesn't knock my tower down", and she'll look at you and smile and make her way over, cruising or take a couple of steps, she's getting better about not leaning into the box, and then get over and knock the tower over. And then we build it back up, and she can help us try build it, but normally we build it, and she knocks it over. And with a big “Oh-Oh” or “Boom”.

You know I try when working on sitting, I will try to create some sort of environment in the house where the parent doesn't have to be behind the kid. I mean I'm always behind them, but they don't care because I’m the PT, so it doesn't matter as much. But for the parents, I had one parent say like “Oh my god, I never get to look at her face when I’m playing with her”, because she was always behind supporting her. I try to explain to parents, the balance between giving them time in a supported environment to do other kinds of play, and then working on that individual skill. If you're always trying to work on sitting and play while they're in sitting, they can't play when they're just trying to stay up. I try to explain that to parents, because some parents don't want

to just stick them in a highchair, then they are not working on sitting. But if they realize that they like to sit up, then it can translate to being more motivating on the floor to want to get up. And then there is also, sometimes I’m like why don't you give them more support on the floor and let them play with the toy. Instead of 'look they can hold it for 3 seconds', and I’m like 'that's great, but what are they doing?' You know they're frozen, they can't even observe what's going on. There's such a balance.

The play needs to be geared towards the goals that you are working on, and you have to figure out how to get the kids to play in a way that makes it therapeutic.

In relation to her, who has her challenges with spasticity, I think we need to stretch, but how can we stretch without her resisting it? I try to find positions where she can play. But then it's perhaps not play, but more of a diversion.

When it comes to her spasticity, how can we stretch without her resisting it? I try to find positions where she can play [while I stretch]. I have this sort of rattle box with lots of things she can explore, and she's really interested in it. And I see that when she opens it, there's a lot she wants to grab. And there are things in there that develop the grip in different ways. But yeah, it's then the way I give her the toy, vertically or horizontally, cross grip. So, you're conscious of the way you offer something, offering a ball that fits right in her hand so she can open her whole hand, if that's the grip I'm after. Or I choose a toy that can roll on the floor if I want her to crawl.

I want to find something that they like, I don’t ask them to do a game they don't want to do. But if they are cognitively impaired, they might be agitated, because they don't know what to do (...). But I'm like ‘We're gonna do it (...) I don't mind a little agitation’. And we work through it, and maybe at the end it is joyful.”

How to play in physical therapy – meaning units

Joining in on play

That's my strategy for this age, I lay out the stuff and then allow them to choose what they might want to do.

I try to have plenty of time. So it's very often that I give up a bit on my project, and then I join in the play. I still have my agenda in the back of my mind, though, and I often find that I can change the play to something that is more up my alley. But you need to give it time. I can't just come in and say ‘now we have half an hour’ and then we do this and that and that.

For the most part, I'm following the child's lead and try to be creative and come up with ways to work with the child and still attain some therapeutic goals

He also fetches the rabbit and the bottle and puts them in, as his mom says, that's what he drives around at home. There's no doubt that here he has a plan and an activity going that's important to him.

He's been a lot into his own things, and then I've been able to move things around and sometimes he's followed them and sometimes not. So, he's a bit in and out of that role-playing, but it's better than it has been. I'm trying to create some interaction.

I can't keep it up for too long. With others, maybe I could have continued, right, but I lose him a bit, there he's seen something else, and there I've lost him again.

It is child directed, so if the kid is playing with a tea-bag, you're gonna first try and make that play, but you're gonna go back and forth, putting it in the box etc. And if not, let them do what they're gonna do and try to incorporate what you can. If you go in with a plan it's gonna go out the window in two seconds. I don't think it gets in the way, you can't go in there like this is how we're gonna play, you have to see what the kid is doing and then try and play with them. You have to do what they are playing with and then expand on that a little bit.

He was barely 6 months when I said we got to get this kid a seating system, cause mom was saying he doesn't want to lie on the floor. But he doesn't have enough strength to do any kind of supported sitting. And if he is, all he is focusing on is sitting, he cannot be upright and engage with what was going on around him. And that was becoming very frustrating for him, and something that mom mentioned almost every session. He doesn't like to lie down, he can't see what's going on, he gets mad, but if he wants to be up the only way is on our lap and we have to do all the work to pull him up. So, they can't play with their kid because they have to hold him up.

I've caught myself joking about it a couple of times, that I realize that I'm not working towards a goal, it's not therapeutic anymore.

When play and therapy get in each other's way

I see that we use a lot of play, or we have to when we work with children, it has to be playful. But it's a bit ambivalent too, because sometimes I feel it can become a bit like 'nonsense', a bit general. There's a bit of ambivalence regarding that, because I want to work specifically, but at the same time, I see that I obviously have to work through play. But to get to work on what you want, to work with quality in movements and such.

So it's like, what's my role in this, should I be the one who has the interaction, or should I be the one trying to work with arms and legs? So interaction is kind of left to the assistant. I could maybe have set up the play so that he was facing me and had contact with me, but I was quite satisfied there too, I think that my task is to try to create good starting positions for him.

I got excited on his behalf you know, and then play disappeared and there was just movement, without much play.

Here, it's important that it should be on the child's initiative, you need to find what makes the child want to put in the effort. A joint project, enjoyable, play, motivating, yes. It's an important part of childhood. I think therapists - and physical therapists - should be better at using movement pleasure and play rather than focusing on the correct movement patterns.

It's absolutely crucial, but it's a balancing act. At the same time, it places quite high demands, something I've learned a bit with experience now, the educational ability when you're working with play, because if you let the kid control everything, then the kid chooses what he's confident in and what he already knows. So, there is this balancing act where you want to challenge him, in a nice way, through play. And every now and then I lose some there because they, I can't get them involved in things they feel is difficult. So it's constantly milling around, that pedagogy in it and how you should approach to achieve what you want.
I look at how far we've come, what activity I want to introduce, where I can build in a bit more of a challenge and such things. But then the child is not interested at all, that's very often the case. So, my project collides with that of the child.

It just triggers something in me, because I have a bit of ambivalence there where many times. I feel that, especially when we're out advising in kindergartens and such, it becomes a bit like 'yeah so you can create an obstacle course, singing games - it sometimes gets a bit unspecific. But we don't really have other options. I've tried, maybe with slightly older kids, to have fixed exercises you do. Then I see, okay, it maybe works for two weeks, and you have to really work to keep up the motivation, but then you can't sustain it over a longer period.

The advantages are that you can get volume. The disadvantages are whether you get the quality in the movements as you want. You have to accept a bit more compensation, because then you're thinking more about function too.

We use toys and play to elicit a certain activity. But maybe it's not really play, because I have an agenda, and I think that then I'm disturbing the genuineness of play. Just like when you intervene in or interrupt a role-play. That the adult mistakenly steps in and directs, consciously or unconsciously, how to play. And then I think that's probably what happens with infants as well.

Because we use a lot of toys. So, there is this risk that it becomes a bait, and then the child gives up when they can't do it, right? And if the child constantly experiences not being able to do it, then I think that's negative in therapy. So, we need to let them have the chance to grasp, it shouldn't just be using it as bait and then taking it away, and then use it as bait a bit more and taking it away again. Because then they kind of resign. And some kids resign very fast. So, I try to be aware of that, when I offer a toy, that they get to explore and study it, turn it around, i think that it's important.

There's also an inner dilemma when it comes to play, and the concept of play, right? Some think that play is only something that happens when you haven't planned a goal. That play only occurs in the moment and arises right then and there, without any intention of achieving something. Well, the play often has a goal within itself, let's say we want to build a tower. But then I add some external goal, that's when it starts to get troublesome. It's a joint project where I have my intentions, but I think it's important that we manage to create something joyful together, that the child feels ownership of and wants to keep on doing.
And then it's crucial that I manage to interpret it correctly, that was what you wanted, right? But we're not really equal, so can we still call it play?

So, I'm thinking, if he just lies there stretching, maybe that's not therapy? The problem is, when he tries to do it, he loses his position. I would gladly let him do it if he had the skill, if he was able to use a single elbow support then I could have let him reach toward himself in the mirror, right. But it was too difficult. I could have also told the mom to bring the mirror closer, but that might get a bit too close then.

Play is hard

You just have to roll with it (…). I think the fact that it's so unstructured and so unpredictable is what makes it entertaining, and keeps you challenged. (…) It was hard when I started out doing it that way, but the benefit is that you're being challenged more, you're thinking outside the box

As long as the child has their own drive and we manage to find things they like, I experience that it's more successful. Or having a joint project, like now we're going to feed the fish, can you bring the fish food?

I find it most challenging with the children with whom it is difficult to interact. Or those children where it's difficult to find a type of play that they can master. Where maybe an iPad is what they can handle. And that can be fine for periods. But the interaction, the development together, I think it's a big loss the way I see it. Being in interaction with others, seeing and exploring. And some have no interest in anything, nothing I've figured out anyways.

Sometimes it just doesn't work. and then I try and figure out why. Is it our chemistry? Is it about me? Is the child out of shape, or didn't want to, or was it just the wrong day? Or is this an insecure child, or what's it about then? It's not always easy to analyze.

I often feel that I succeed, to be completely honest. It was much more difficult when I was a fresh graduate. Now I have a larger repertoire to play with, so I can just change. Change my role or focus in play or type of play, to get where I want.

There are many questions. What kind of play, how does play work in different settings, what role does the therapist take in relation to the child, in relation to what we want to achieve? I'd like to become more aware of how I use play. I think I end up doing a lot of the same thing, it quickly turns into a routine. So I want to be a bit more conscious of how I use play, then maybe I can get the kids to do what I want, to put it bluntly.

No, where you have less drive, and there's poor eye contact I would say. But then it's almost like I have to ask you if you have any tips on how to facilitate play? But I think that, when it comes to the cognitive, you have to think about how you would have played with a healthy child, right, how you would have stimulated them, even though you see that the response isn't the same.

First, it was about capturing his interest, and I don't see him make any good eye contact. So, there I thought we are very far from getting his arms up. Then I try with two different toys and see, trying to make a sound, that's my first goal to capture his interest. Then you see, he does catch a bit with his legs. But there's little response in the arms, and it's not an active look either. Such a fixed gaze, if I move the toy, the head doesn't follow. He's making it quite clear that there are several things he's not ready for yet. And I try to take that in, that the play interest is just not there. It's a valid point, if he's not interested in the play and we're working towards getting him to come and grab the toy. But at the same time, I see that when we lead him into that grip, he moves it towards his mouth a bit, so he's kind of on his way.

Like when we were dealing with the food, because I haven't managed to get much role-play going with him. I've tried a lot and then I tried giving him food and ice cream, yogurt, and then I managed to engage him a bit. But here too, I'm thinking a bit about the cognitive. But we don't know, he's under evaluation. He's 19 months, but I've never heard him say mom, dad or mommy and daddy.

When a kid isn't able to expand on their play skills. I do feel that it is ok to meet a kid wherever they are developmentally. But when they can't expand upon that, to be able to really progress their gross motor skills, it becomes challenging.

If you can't manage to play with that child, and I would actually say that I have quite a lot of experience with playing, I do a lot of that, and then I always ponder why this child isn't participating. Maybe trying a lot of different things. Then I've seen in hindsight, some might end up with an autism diagnosis. It rings quite quickly, why aren't they participating, most kids let themselves be fascinated by an adult who is really into the play and wants to play. So it starts to churn quickly if they're not on board with that.

My experience in relation to whether they receive a cognitive diagnosis is that there's a reason we also see them, because their motor skills are inhibited by it, subsequently. But clearly, it's connected, if you're not interested in playing with other kids or don't care much about toys, aren't fascinated by them, then that affects the motor skills subsequently, leading to less variation, and they move less which causes delays.

It's very difficult, I find it one of the hardest things to work with as a pediatric physiotherapist, when it's autism, and then the kindergarten reports that he has poor balance, but how are you supposed to work with that? It's extremely difficult, and I still struggle with it. I often end up with, say a 2-year-old, giving advice about, right, a normal 2-year-old, where is that? Can you somehow manage to work with that? But it's no use if I come once a week to the kindergarten and think that I'm going to achieve it. I had a 3-year-old now, for example jumping with feet together, not even close, but how you're supposed to work with that concretely when you have autism, for example.

He's a bit like if you're too engaged, and take him too much out of the play, then he protests, then he clearly indicates that this wasn't what he had in mind.

Sometimes I notice that some kids seem to have very little initiative for a while, and then it's like they wake up at some point, and a lot happens. And then I really wonder what made them suddenly get that ability to take initiative and move forward, the curiosity wakes up. Clearly, the more you can do with your body, the more you might want to try out more stuff. And both these boys are examples of having a lot of things that disrupted their development early in life. There have been illnesses and stuff that had to be handled. Just think how robust the system is, though, when they suddenly can bloom and catch up on things that were lagging behind. They're good examples of the recovery ability that's actually there.

I figured, the way I see it, he's really focused on this cup, that's where he wanted to be. He didn't jump on what I was thinking, like maybe getting him on all fours. He wanted to lie on his side and play, right. So, I tried to get him started with something more, but he wasn't having it, not right away at least. So, I end up stopping the play and saying, no, I want to move on. I get eager, you know, on his behalf, and then the play kind of faded away. And then it turned into movement without much play.

I think the most challenging are children who are functioning at a very low level. You know trying to have them find ways to access play activities can be very challenging. You know the cognitive piece of figuring out what's going to motivate them, and then the motor piece of figuring out how they can access whatever it is we find.

I think it's a lot of the personality of the kid you're working with. The kids who are not motivated to move are the hardest kids for me to work with. Because there is nothing, no play entices them to do what I want them to do.

A lot of our sessions was more like catching up and talking, and what's going on and tell me what you're doing, and mom will try to do stuff and a lot of times with mom he wouldn't engage. kind of like the pull-to-sit, he was like no. That used to be most of our sessions as he would have a hard time. Toys are still a new thing, but social has also not been easy. So, I think that was the biggest thing, all his engagement with a toy, his tolerance. The fact that he was finding things motivating enough for him to want to try to do something that is hard, that was big. and new. Q: so that is the essence that I'm hearing, now you see him start to play, for real? A: Yes. Not just studying this new thing or just watching the world around him or watching his sister run and jump or whatever, but yes, he's really engaging with toys and playing with toys, and independently motivated to play with the toys. Not just having somebody do it for him.
